# Age- and Sex-Related Changes in Labial Dimensions of Sudanese Youngs of Arab Descent: A Three-Dimensional Cross-Sectional Study

**DOI:** 10.3390/children8070574

**Published:** 2021-07-04

**Authors:** Claudia Dolci, Fadil Elamin, Daniele M. Gibelli, Luisa Barni, Alessandra Scolaro, Fabiola Sessa, Cinzia Maspero, Annalisa Cappella, Chiarella Sforza

**Affiliations:** 1 Laboratory of Functional Anatomy of the Stomatognathic System (LAFAS), Department of Biomedical Sciences for Health, Università degli Studi di Milano, 20133 Milan, Italy; daniele.gibelli@unimi.it (D.M.G.); luisa.barni@unimi.it (L.B.); annalisa.cappella@unimi.it (A.C.); chiarella.sforza@unimi.it (C.S.); 2 Khartoum Centre for Research and Medical Training, Khartoum 11111, Sudan; fadilelamin@yahoo.co.uk; 3 Institute of Dentistry, Barts and The London School of Medicine and Dentistry, Queen Mary University of London, London E1 2AD, UK; 4 Department of Biomedical, Surgical and Dental Sciences, School of Orthodontics, Università degli Studi di Milano, 20122 Milan, Italy; alessandra.scolaro@unimi.it (A.S.); fabiola.sessa@unimi.it (F.S.); cinzia.maspero@unimi.it (C.M.); 5 Department of Biomedical, Surgical and Dental Sciences, School of Dentistry, UOC Maxillo-Facial Surgery and Dentistry, Fondazione IRCCS Ca Granda, Ospedale Maggiore Policlinico, 20122 Milan, Italy; 6 UO Laboratory of Applied Human Morphology, IRCCS Policlinico San Donato, San Donato Milanese, 20097 Milan, Italy

**Keywords:** anthropometry, 3D, lips, growth, Sudanese, Arab descent

## Abstract

Proper evaluation of facial features during growth and development requires the knowledge of anthropometric reference values validated for ethnicity, sex and age. In order to provide information concerning the normal sex-related size of the lips during childhood and young adulthood in Sudanese people of Arab descent, the three-dimensional coordinates of nine labial soft tissue landmarks were obtained by a laser scanner in 332 male and 386 female healthy Northern Sudanese subjects aged 3–30 years. Six labial linear distances, the vermilion height to mouth width ratio, vermilion areas and lip volumes were calculated and averaged for age and sex. Comparisons were performed by factorial analysis of variance (*p* < 0.01). All labial dimensions significantly increased with age. Significant effects of sex were found for four measurements only, with very small effect size; nonetheless, lips and their parts grew faster in females than in males at almost all ages. Philtrum width was the first linear distance that attained adult values. The vermilion height to mouth width ratio was nearly constant across the age groups. Data collected in this study contribute to information about ethnic-specific lip morphology during growth and development. As orolabial features change over time with their own pattern, the relevant age-related trends should be properly considered for clinical treatment planning.

## 1. Introduction

Together with our name, our face characterizes ourselves and allows our identification among the hundreds of people we meet every day. A central role in facial assessment is played by the mouth and lips [1,2,3], which also convey information about sex, age, ethnicity, health conditions, mixing function, esthetics and social aspects in unique configurations [1,4,5,6,7,8].

Healthy facial structures clearly contribute to the global well-being of people of all ages and geographical origins and their restoration after local or systemic disorders goes well beyond clinical aspects. A detailed knowledge of the normal characteristics of the mouth and lips is usually provided by anatomical and anthropometrical studies; the relevant reference data are employed in a variety of medical fields, from reconstructive, surgical and dental treatments to genetic investigations of the facial phenotype for early detection and diagnosis of diseases involving the face, as well as facial reconstruction for forensic purposes [1,3,4,8,9,10,11,12,13,14,15,16,17,18,19,20,21,22]. 

In particular, to better identify those individual features that best discriminate among people, those that are more or less variable in life, age-, sex- and ethnic-specific databases should be provided: this information is crucial for treatment planning and the timing of dental, orthodontic and surgical therapies [1,3,5,6,9,13,16,19,21].

Currently, investigations may be performed using two-dimensional photographs, but methods to solve scale and calibration issues are required, as well as rigorous standardization of photographic procedure [23]. Optical image analysis systems working in three-dimensional (3D) space allow us to overcome that limitation; among them, stereophotogrammetry, laser scanning and structured light cameras are probably the most used worldwide, coupling minimal or null patient discomfort with a fast data collection [2,8,10,11,12,18,24,25,26,27,28,29,30,31,32]. In addition, intraoral dental scanners have been recently successfully proposed for the digitization of the superficial soft tissues of the nasolabial area [4]. 

Dimensions, relative proportions and reciprocal spatial positions of the structures of the orolabial region in healthy subjects of different ages have been widely investigated. Unfortunately, most of the studies report data for Caucasoid and Chinese subjects and investigations about Africans/African Americans are few and mostly limited to adults [1,3,5,6,8,9,16,18,19,21,25,31,32,33,34,35,36,37,38,39,40]. The lack of information about normal orolabial structures is one of the problems that contribute to an unequal distribution of resources among countries, thus preventing the access to diagnosis and tailored treatment planning. 

Among studies concerning African subjects, some recent investigations reported data about people from Sudan [3,9,19,31,32]. Sudan is located in the northeastern part of Africa; it is one of the largest African countries. Overall, Sudanese people have mixed ethnicity, as they descend from African, Nubian and Arab tribes, with the latter the most represented in the population (approximately, 70%), with additional contributions from Middle Eastern populations [9,19]; each ethnic group shows its own facial appearance [30]. At the beginning of 2011, the population of Sudan was estimated to be about 33.4 million people, with a growth rate of 2.53% annually [41].

Up to now, the literature concerning the craniofacial district of healthy people from Sudan has mostly focused on people of Arab descent and has illustrated the dental arch characteristics [39] and the general soft tissue facial dimensions of healthy subjects [9,19,31], reported detailed information about the facial upper third and orbital area [30], described the facial profile and its soft tissue thickness [3,32], and investigated the presence of neoclassical canons of facial harmony [19], but in no occasion has a comprehensive analysis of the orolabial region been provided. 

In particular, both Sforza et al. [32] and Hamid and Abuaffan [3] reported that the soft tissue profile of the middle and lower third of the face of Sudanese children, adolescents and young adults had different dimensions and thickness than in other ethnic groups, being somehow midway between Caucasoid and African reference values from the literature. Furthermore, peculiar growth patterns were observed for some measurements [32]. 

Therefore, improving knowledge of the characteristics of the orolabial area in Sudanese people during growth and development seems necessary as their facial features are potentially unique among the other African ethnicities. 

Accordingly, the aim of the current study is to provide a detailed analysis of the normal sex-related dimensions of the mouth and lips during childhood and young adulthood in Sudanese people of Arab descent. Linear distances, areas and volumes concerning orolabial area were evaluated non-invasively using laser scanning. 

## 2. Materials and Methods

### 2.1. Subjects 

A total of 682 (332 males, 350 females) healthy Sudanese subjects of Arab descent from the northern part of the country, aged 3 to 30 years, participated in the study. Parts of the collected data were previously used as reference values for the calculation of z-scores in the analysis of oronasal morphology of Northern Sudanese subjects with Down syndrome [32]. The subjects were divided by sex and into nine non overlapping age groups as described by Sforza et al. [40]; 2-year spans were used for subjects under the age of 18 years, while a larger interval was used for young adult subjects. Each sex and age group comprised a minimum of 29 subjects, except for the youngest subject group (13 females and 6 males, respectively). No significant age differences were found between the two sexes (Student’s *t* test, *p* > 0.05). 

All subjects participating in the study were in good health and did not suffer from any major systemic disorders; they had no previous history of craniofacial congenital anomalies, traumas or surgery. Furthermore, their occlusion was considered harmonic at visual inspection, without excessive inclination of the incisors. 

They were informed about the experimental procedures and gave their consent to the study. For children and adolescents aged less than 18 years of age, informed consent was obtained also from their parents/legal guardians. All procedures were not invasive, not harmful and did not cause discomfort to the subjects. The study protocol was approved by the local ethic committee (Ethical Approval Committee at Elrazi Dental School, Reference number: Dent/01). 

### 2.2. Collection of 3D Facial Landmarks

**Figure 1:**
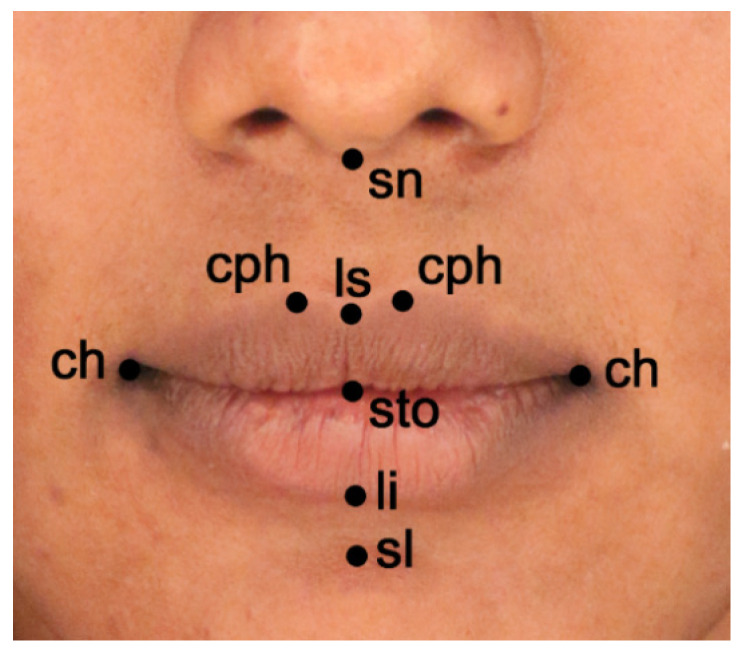
Digitized 3D labial landmarks used in the current study (sn, subnasale; ls, labiale superius; sto, stomion; li, labiale inferius; sl, sublabiale; cph, crista philtri; ch, cheilion).

The same data collection procedure from our previous investigations was used, as detailed elsewhere [31,32,42,43]. By using a portable hand-held laser scanner (FastSCAN Cobra; Polhemus Inc., Colchester, VT, USA), 3D facial surface images of the subjects were recorded. A single operator acquired the facial image of all the subjects. Offline, the facial models were visualized by using an interactive 3D modeler software (Rhinoceros NURBS modeling for Windows 4.0) and the x, y, z coordinates of a standardized set of 50 soft tissue landmarks were obtained. Subsequently, custom computer programs were used for anthropometric measurements [32,40,43,44]. A subset of the collected landmarks was employed in the current study (Figure 1): sn, subnasale; ls, labiale superius; sto, stomion; li, labiale inferius; sl, sublabiale (midline unpaired landmarks); cph, crista philtri; ch, cheilion (on the right-r and left-l side of the face or paired landmarks). 

As previously reported, the accuracy and precision of the laser scanner were tested and were found to be adequate for facial anthropometry [32,43]. In detail, reproducibility of landmark identification and facial digitization was tested in 10 subjects. The set of 50 landmarks was identified and digitized by the same operator on two occasions, 1 month apart; 18 linear distances were then calculated. Repeated data collection sessions were found to be free from statistically significant systematic errors (Student’s *t* test for paired samples, *p* > 0.05), with a mean random error (technical error of measurement, TEM) of 0.755 mm. 

### 2.3. Data Analysis 

The 3D x, y, z coordinates of the nine landmarks digitized on each subject were used to compute a set of linear distances, surface areas and soft tissue volumes, by applying the geometric model of the nasolabial area defined by Ferrario et al. [44]:

linear distances (unit: mm): mouth width (ch_r_-ch_l_); width of the philtrum (cph_r_-cph_l_); vermilion height of the upper lip (ls-sto); vermilion height of the lower lip (sto-li); total vermilion height (ls-li); total (cutaneous) lip height (sn-sl); the vermilion height to mouth width ratio was subsequently obtained (ls-li/ch-ch x 100, unit: %);

areas (unit: mm^2^): vermilion of the upper lip from the quadrangle between ch_r_, ls, ch_l_, sto); vermilion of the lower lip from the quadrangle between ch_r_, li, ch_l_, sto); total vermilion from the quadrangle between ch_r_, ls, ch_l_, li;

volumes (unit: mm^3^): upper lip volume from the volumes of two tetrahedra: (1) base between ch_r_, ch_l_, ls; vertex in sn; (2) base between ch_r_, ch_l_, ls; vertex in sto; lower lip volume: (1) base between ch_r_, ch_l_, li; vertex in sl; (2) base between ch_r_, ch_l_, li; vertex in sto; total lip volume (sum of the four tetrahedra).

### 2.4. Statistical Analysis

Descriptive statistics (mean and standard deviation) for each measurement were computed separately for sex and age groups. Mean values between sexes and age groups were compared through two-way factorial analyses of variance. The effects of sex (two levels, males and females) and age (nine levels, from 3 to 30 years of age) were assessed, together with the sex x age interaction. Considering the reduced number of landmarks and the interrelated measurements, significance was set at 1% (*p* < 0.01), with two-tailed statistical tests used in all analyses. The Free Statistics Software (Office for Research Development and Education) was used [45,46]. For significant effects and interactions, the partial η^2^ value was computed to estimate the effect size, that is the practical importance of the statistically significant difference. Partial η^2^ values lower than 0.02 are considered very small, up to 0.13 small, between 0.13 and 0.26 medium and from 0.26 to 1.00 large [47]. 

To identify the various growth patterns before the attainment of skeletal maturity and to assess the developmental level of each measurement, the analyzed distances, areas and volumes were expressed as a percentage of the mean value obtained in the last age class (young adults, 18 to 30 years of age), and descriptive statistics for each age group and sex were computed [31].

## 3. Results

All labial volumes, areas and linear distances significantly increased from childhood and adolescence into young adulthood; all partial eta squared values were larger than 0.174 (medium to large effects, Table 1, Table 2 and Table 3). The larger age-related differences and partial eta squared values were observed for vermilion areas and lip volumes, as well as for mouth width. The mouth height to width ratio also showed a significant effect of age, but with a small partial η^2^: the ratio was almost constant after 4–5 years of age. 

Statistically significant effects of sex were found for four measurements only: total and lower vermilion area, mouth height and lower vermilion height. However, their corresponding partial η^2^ values were very small. Only for a single anthropometric measurement, namely the width of the philtrum, was the age x sex interaction significant, with a small effect size: it was almost always larger in females than in males up to 12–13 years of age, while the opposite was observed in the subsequent age groups.

**Figure 2:**
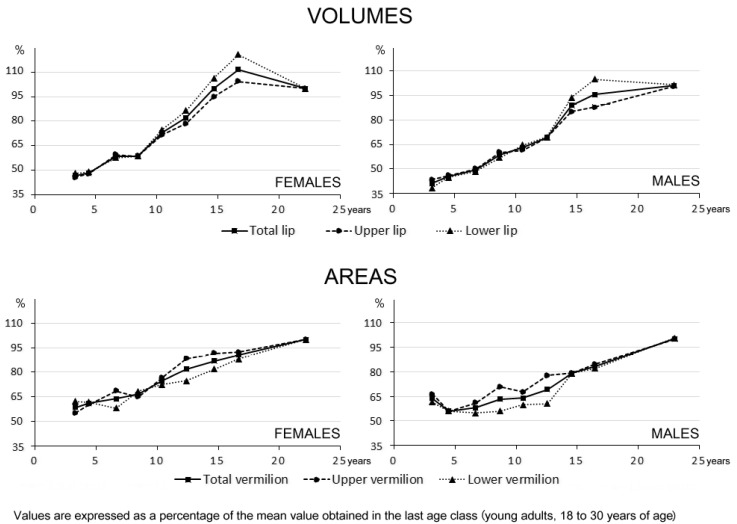
Age-related variations of soft tissue lip volumes and vermilion areas in females and males.

**Figure 3:**
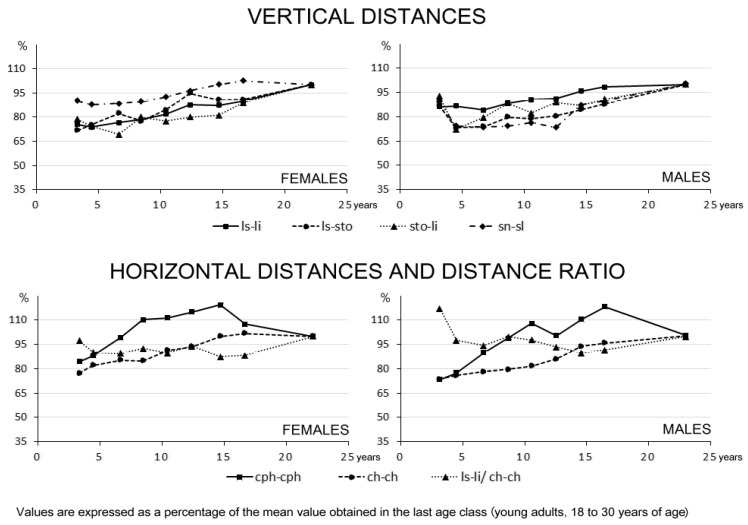
Age-related variations of soft tissue lip distances and distance ratio in females and males.

Figure 2 and Figure 3 show the age-related modifications of the analyzed measurements in the two sexes. Data are expressed as a percentage of the attainment of adult dimensions; values greater than 100% may be accounted for in the cross-sectional design of the study.

Concerning lip volumes and vermilion areas (Figure 2), at almost all ages females grew faster than males and attained more than 90% of adult values in the 14–15-year-old age group (volumes) or in the 16–17-year-old age group (areas). The effect was more evident for the lower lip volume and for the upper vermilion area. 

In both sexes, vermilion areas grew faster than lip volumes during childhood, while the pattern reversed after 10 years of age. The vermilion areas were surprisingly larger in the first male age group than in the two subsequent ones. It must be pointed that the number of analyzed boys was limited in that age group; furthermore, outlier values were observed.

The sex-related growth patterns of linear distances (Figure 3) were similar to those seen for areas and volumes: female values (as a percentage of adult values) were almost always larger than male values of the same age group. 

The philtrum width was the first linear distance that attained adult values: mean percentage values larger than 90% were reached at 6–7 years of age in females and 8–9 years of age in males. Among the other linear distances, in both sexes the total (cutaneous) lip height reached 90% of its adult value in the 10–11 year old age groups. Mouth width had a similar trend in girls, while male growth was slower, with attainment of more than 90% of adult values at 14–15 years of age. 

Consistent with the vermilion volumes and areas, the vermilion heights reached approximately 90% of their adult values in the last adolescent age groups of both sexes, its growth being faster for the upper than for the lower lip. 

As described previously, the vermilion height to mouth width ratio was nearly constant in the analyzed age groups, with small differences compared to the adult values. 

## 4. Discussion

In healthy Sudanese subjects of Arab descent, linear distances, areas and volumes of the orolabial area have been found to change significantly between childhood, adolescence and young adulthood. Sex-related values seem less important in children and adolescents of the current ethnic group. Nonetheless, even if the mean values of most of the analyzed measurements did not differ significantly between males and females, their growth patterns were sexually dimorphic and female percentage attainment of adult values was almost always larger than that obtained by males of the same age group. 

To the best of our knowledge, no other investigation has reported comparable data, especially in children. Sforza et al. [31,43] described some soft tissue facial distances and analyzed a set of midline angles and distances from Ricketts’ aesthetic line, finding several differences related to data collected in African and Caucasoid subjects. A detailed definition of the normal dimensions of facial structures in different ethnic groups, ages and sexes is therefore essential for a correct reconstruction of the global facial appearance in the forensic field (facial aging, facial reconstruction from skeletal remnants), in patients (via orthodontics, dental prostheses, surgery) and in the design of medical devices [1,3,8,16,18,25,39]. In particular, taking the esthetic characteristics of the mouth area into consideration, proper relationships between the cutaneous and vermilion parts of the lips should be maintained or restored by surgical and dental reconstructive treatments, thus contributing to the general well-being of people [5,6,22].

In the current investigation, the same age groups chosen in a previous study on healthy Italian Caucasoid children and adolescents were used [40]. Indeed, the previous study reported data from 3 to 73 years of age and the two investigations are only partially comparable: even if the same measurements were made, there are different ethnicity and age spans and different data acquisition protocols. In the Italian study, several variables were significantly influenced by sex (labial volumes and total vermilion area, mouth and philtrum widths, total lip height and mouth height to width ratio) while in the current study only four of them reached the statistical threshold, but with very small effect sizes. Only the total vermilion area and lip height were sexually dimorphic in both studies [40]. 

Notwithstanding the biological and technical differences between the two studies, a similar general age-related trend was observed, with males growing for a longer time than females. Furthermore, several measurements were comparable between the two ethnic groups, especially in males: the total lip volume, total and lower vermilion areas, and total lip height were similar, as well as the mouth height to width ratio. However, upper lip volume was larger in the Italian than in the Sudanese subjects of the same sex and age, while the opposite was observed for the lower lip volume, thus differentiating the two ethnicities [32]. Furthermore, the upper vermilion area was larger in the Italian children and in the Sudanese adolescents relative to their sex- and age-matched peers. A similar trend was observed for the upper vermilion height in females; it seems that vermilion growth begins earlier in Italian than in Sudanese subjects. Finally, the mouth and philtrum widths and the total and lower vermilion heights were similar among males, but wider and longer in Sudanese than in Italian females [40]. 

The mouth height to width ratio was significantly modified by age in Sudanese subjects but, even when statistically significant, the difference was practically negligible. This ratio may be used when a relatively constant measurement is needed: in both sexes it reached 90% of its adult value in the first age groups. Among the linear distances, a similar pattern was observed for philtrum width. In contrast, vermilion area and height showed a nearly constant increment during the analyzed time span, especially for the lower lip. The global dimensions of the labial vermilion may help in differentiating the various age groups. 

A recent photographic study, performed in an ethnically mixed Sudanese group of healthy young adult women, found an average mouth width that was approximately 7 mm smaller than the current value [19], narrower than most data from the literature (Table 4). As a matter of fact, even if the measurements shown in Table 4 also differed for technical aspects, as diverse data collection protocols were used, the biological variety played an important role. For instance, the average mouth width of African American women is about 7 mm wider than the value of Egyptian women, where both data collections were performed using calipers [33,34]. The same difference can be observed for men (Table 5), thus supporting the presence of actual ethnic differences between Egyptian and other African/American African young adults. The wider mouth width was found in Zulu men, with a clear sexual dimorphism, while the narrowest values were reported for the Italian subjects, in both sexes. 

Concerning the other linear distances measured in the current study, the literature is scant. Overall, the current philtrum width was wider than values collected in both African American and Caucasoid young adults, while the vermilion heights were longer than in the Caucasoid subjects and shorter than in the African American subjects (up to 7 mm). In the latter case, a technical difference may partly explain the result, as the current values, as well as those reported for the Italian subjects, were obtained with a computerized geometrical model while those listed by Farkas et al. [33,48] resulted from caliper measurements that were summed. The total cutaneous lip height was similar to the Italian values.

Recent 3D comparisons between digital models of the faces of healthy young adult men and women of American Caucasoid, African American and Zimbabwean origins outlined a set of specific differences, that, both between and within ethnic groups, mainly focused on the nasolabial area [8,10]. A cephalometric analysis showed that Sudanese people possess a specific pattern of soft tissue facial thickness that differentiates them from African Zulu, Iraqi and Brazilian people with similar maxillomandibular horizontal skeletal relationships. Furthermore, the pattern had sex-related differences [3].

Indeed, as far as mouth width is concerned, Fang et al. [13] found that its inter-ethnic variability was only intermediate. It has to be mentioned that their data were limited to adult subjects aged 18 to 35 years and that only 4 out of 27 ethnic groups were African or African Americans, none from Sudan. Therefore, it seems that Sudanese young adults have a unique facial pattern, different from that found in persons of African and Arabic origins [3]. 

Overall, we can combine the current results with the more recent and complete analyses of the orolabial area describing soft tissue thickness [3], dimensions and relative positions of the lips and nose [9,19,31,43] and dental arch characteristics [39] to provide a dataset to be used for clinical and forensic applications with Sudanese people.

## 5. Limitations 

Some study limitations should be considered. All the analyzed measurements significantly changed as a function of age, but the current data were cross-sectional, with different groups of subjects examined at different ages [25]. Actual values do not reflect the real growth but are estimates of the biological phenomena. This a major drawback of cross-sectional investigations: the observed age-related differences may be an effect of secular trends. Nonetheless, the current differences were in good accordance with results shown by longitudinal reports performed in pre-school and adolescent boys and girls [20,27,29].

The youngest age classes comprised a small number of children and their data should be considered with caution. Furthermore, some of the partial eta squared values were small and the practical use of the relevant measurements should be attentively considered. At the same time, only subjects aged 3 to 30 years were examined and the data collection should also be performed after the third decade of life. Furthermore, subjects with different ethnical descent should be analyzed, thus completing the description of Sudanese people.

A harmonic occlusion without an excessive incisor inclination was verified by visual inspection in all subjects participating in the study, with the position of the lips influenced by the presence of malocclusion [3]. A more objective evaluation might have been useful but neither invasive radiographs to assess skeletal class occlusion nor the assessment of dental angle class were performed.

From a technical point of view, a hand-held laser scanner was used for data collection: facial scanning needs approximately 20–30 sec and motion artifacts may be possible [26]. Indeed, this is a limitation common to most of the hand-held instruments, especially when a laser beam is used [28]. Nonetheless, the instrument and the data collection protocol have been reported to be clinically acceptable [32,43,49,50]. 

## 6. Conclusions

The labial dimensions of healthy Sudanese subjects of Arab descent have been found to significantly increase during growth and development, with a limited sexual dimorphism in absolute values, but with different age-related patterns in the attainment of adult sizes between the two sexes. 

Linear distances, areas and volumes in the orolabial area change with their own pattern during childhood and adolescence. In particular, the dimensions of the labial vermilion undergo a nearly constant increment; their evaluation may help in differentiating the various age groups and their age-related trends should be properly considered for clinical treatment planning. On the contrary, the mouth height to width ratio, as well as the philtrum width, change over time with practically negligible differences and they could be used when a relatively constant measurement is needed, such as for personal recognition.

Data collected in the present study could serve as a database for the quantitative description of mouth and lip morphology in Sudanese people of Arab descent during normal growth and development, thus filling one of the current literature gaps. 

We trust that the current investigation can be one further step toward the achievement of the WHO goal of “health for all and all for health”, offering medical and dental professionals information to promote health in Sudanese people both in Sudan and abroad.

## Figures and Tables

**Table 1:** 3D soft tissue labial volumes, areas, distances and ratios in healthy Sudanese females of Arab descent.

Females	Age	3 Years		4–5 Years		6–7 Years		8–9 Years		10–11 Years		12–13 Years		14–15 Years		16–17 Years		18–30 Years	
	N	13		41		32		30		46		42		29		42		75	
Measurement	Unit	Mean	SD	Mean	SD	Mean	SD	Mean	SD	Mean	SD	Mean	SD	Mean	SD	Mean	SD	Mean	SD
Total lip volume	mm^3^	2615	460	2710	597	3291	918	3287	1070	4094	1005	4605	1281	5617	1360	6269	1790	5616	1851
Upper lip volume	mm^3^	1423	274	1493	356	1856	546	1833	620	2236	668	2452	768	2969	780	3264	888	3128	1007
Lower lip volume	mm^3^	1192	284	1217	318	1434	488	1453	513	1858	433	2152	638	2648	704	3005	1042	2488	962
Total vermilion area	mm^2^	299	41	311	53	325	82	340	67	382	71	418	77	444	96	463	110	510	98
Upper vermilion area	mm^2^	147	29	161	36	183	54	173	41	204	58	235	59	244	62	246	56	267	65
Lower vermilion area	mm^2^	152	22	151	27	143	45	168	53	178	38	184	44	201	60	216	80	245	66
ch_r_-ch_l_	mm	40.6	3.2	43.2	4.1	44.9	4.2	44.6	4.3	48.0	4.2	49.2	5.1	52.6	4.7	53.5	5.1	52.6	5.0
cph_r_-cph_l_	mm	10.8	1.6	11.3	2.3	13.6	2.4	14.2	2.7	14.3	3.0	14.8	2.5	15.3	3.0	14.1	3.2	12.8	3.4
ls-li	mm	14.8	1.5	14.5	2.2	15.1	2.6	15.4	2.9	16.1	2.8	17.2	2.7	17.2	3.5	17.7	3.0	19.7	3.1
ls-sto	mm	7.6	1.3	7.9	1.8	8.7	1.9	8.2	1.7	8.9	2.4	10.0	2.3	9.5	2.5	9.6	1.9	10.5	2.1
sto-li	mm	8.1	0.9	7.7	1.5	7.1	1.8	8.3	2.5	8.0	1.6	8.3	1.6	8.4	2.0	9.2	2.3	10.3	2.3
sn-sl	mm	36.0	3.4	35.1	2.8	35.4	3.7	35.8	3.3	36.9	3.2	38.5	3.7	40.0	4.5	41.0	3.9	40.0	4.7
ls-li/ch_r_-ch_l_	%	36.7	4.7	34.0	6.5	33.8	6.4	34.9	7.5	33.9	7.1	35.5	6.8	33.0	7.6	33.4	6.5	37.8	6.9

SD: standard deviation

**Table 2:** 3D soft tissue labial volumes, areas, distances and ratios in healthy Sudanese males of Arab descent.

Males	Age	3 Years		4–5 Years		6–7 Years		8–9 Years		10–11 Years		12–13 Years		14–15 Years		16–17 Years		18–30 Years	
	N	6		34		37		29		29		32		45		52		68	
Measurement	Unit	Mean	SD	Mean	SD	Mean	SD	Mean	SD	Mean	SD	Mean	SD	Mean	SD	Mean	SD	Mean	SD
Total lip volume	mm^3^	2667	631	2932	967	3189	955	3786	943	4066	1318	4469	1473	5733	1657	6154	1882	6454	1931
Upper lip volume	mm^3^	1550	437	1635	584	1786	493	2141	594	2193	656	2459	852	3024	905	3119	1084	3560	1125
Lower lip volume	mm^3^	1117	299	1297	420	1403	511	1645	492	1872	700	2009	727	2709	881	3035	994	2894	1032
Total vermilion area	mm^2^	365	76	320	86	332	68	361	73	366	62	396	94	452	86	476	111	571	102
Upper vermilion area	mm^2^	190	55	160	48	175	48	203	44	194	40	223	62	227	55	243	66	286	69
Lower vermilion area	mm^2^	175	37	159	46	156	41	159	50	170	44	173	58	225	61	234	60	284	72
ch_r_-ch_l_	mm	41.3	3.3	42.5	6.8	43.8	4.2	44.6	3.6	45.7	5.8	48.2	5.0	52.5	4.7	53.6	6.1	55.9	4.9
cph_r_-cph_l_	mm	10.2	1.2	10.8	2.3	12.5	2.6	13.7	2.7	15.0	2.6	13.9	2.8	15.3	3.6	16.4	2.4	13.9	3.4
ls-li	mm	18.0	3.3	15.1	2.7	15.3	2.8	16.5	3.0	16.3	2.6	16.7	3.2	17.5	2.9	18.2	3.1	20.7	3.3
ls-sto	mm	10.1	3.9	7.9	1.7	8.7	2.3	9.6	1.9	9.0	1.9	9.7	2.9	9.4	2.0	9.9	2.4	10.9	2.3
sto-li	mm	9.7	2.5	8.0	1.7	7.9	2.1	8.0	2.1	8.3	1.7	7.9	1.9	9.4	2.4	9.7	1.9	10.8	2.3
sn-sl	mm	37.1	7.0	37.2	3.4	36.1	3.1	38.0	3.1	38.9	3.7	39.1	4.6	41.2	4.2	42.2	4.2	42.9	4.5
ls-li/ch_r_-ch_l_	%	43.8	7.8	36.4	8.7	35.2	7.0	37.2	7.2	36.5	8.1	34.8	7.1	33.5	6.4	34.1	6.3	37.3	7.0

SD: standard deviation

**Table 3:** 3D soft tissue labial volumes, areas, distances and ratios in healthy Sudanese subjects of Arab descent: effect of age and sex.

Source	Age			Sex			Age x Sex		
Measurement	F	*p*	η_p_^2^	F	*p*	η_p_^2^	F	*p*	η_p_^2^
Total lip volume	61.597	<0.001	0.455	4.341	0.038	//	0.886	0.545	//
Upper lip volume	51.562	<0.001	0.411	4.213	0.041	//	1.103	0.359	//
Lower lip volume	54.557	<0.001	0.425	3.123	0.078	//	0.561	0.810	//
Total vermilion area	66.322	<0.001	0.473	9.044	0.003	0.015	2.153	0.029	//
Upper vermilion area	40.589	<0.001	0.355	1.15	0.284	//	1.804	0.08	//
Lower vermilion area	13.665	<0.001	0.362	11.649	<0.001	0.019	1.835	0.068	//
ch_r_-ch_l_	65.514	<0.001	0.470	1.680	0.195	//	2.268	0.022	//
cph_r_-cph_l_	18.518	<0.001	0.202	0.638	0.425	//	2.978	0.003	0.039
ls-li	31.762	<0.001	0.301	6.51	0.011	//	1.118	0.349	//
ls-sto	15.547	<0.001	0.174	3.233	0.073	//	1.669	0.103	//
sto-li	23.607	<0.001	0.240	7.167	0.008	0.010	1.121	0.347	//
sn-sl	25.033	<0.001	0.253	24.955	<0.001	0.041	0.845	0.561	//
ls-li/ch_r_-ch_l_	4.224	<0.001	0.054	2.991	0.843	//	0.841	0.567	//

Values computed by a two-way factorial analysis of variance; degrees of freedom: sex, 1; age, 8; sex x age interaction, 8. For significant effects and interactions (*p* < 0.01), the η_p_^2^ value was computed.

**Table 4:** Lip dimensions obtained in the current study and in the literature for young adult healthy African or African American women compared to data for Caucasoid women.

	Arab Sudanese [Current Study]		Sudanese Average [19]		AA [33]		Angolan [34]		Egyptian [34]		Zulu [34]		Kenyan African [21]		NAW [48]		Italian [40]	
Instrument	Laser scanner		Photographs		Caliper		Caliper		Caliper		Caliper		Caliper		Caliper		Contact digitizer	
Age (years)	18–30		17–23		18–25		18–30		18–30		18–30		18–30		19–25		18–30	
N	75		100		80		30		30		30		36		200		66	
mm	Mean	SD	Mean	SD	Mean	SD	Mean	SD	Mean	SD	Mean	SD	Mean	SD	Mean	SD	Mean	SD
ch-ch	52.6	5.0	45.7	2.9	53.6	4.0	52.9	5.6	46.7	3.7	52.2	4.0	52.0	4.0	50.2	3.5	48.5	3.6
cph-cph	12.8	3.4			12.0	1.2									9.7	1.5	10.7	2.2
ls-li	19.7	3.1			26.5 ^§^	1.9									17.8 ^§^	2.7	15.8	2.6
ls-sto	10.5	2.1			13.3	1.9									8.7	1.3	6.8	2.2
sto-li	10.3	2.3			13.2	1.9									9.4	1.5	9.1	2.7
sn-sl	40.0	4.7			44.7 ^#^	5.4							44.7 ^#^	3.4	37.9 ^#^	6.8	39.4	3.6

AA: African American; NAW: North American White; SD: standard deviation; ^§^: sum of ls-sto and sto-li; ^#^: sum of sn-sto and sto-sl.

**Table 5:** Lip dimensions obtained in the current study and in the literature for young adult healthy African or African American men compared to data for Caucasoid men.

	Arab Sudanese [Current Study]		AA [33]		Angolan [34]		Egyptian [34]		Zulu [34]		Kenyan African [21]		NAW [48]		Italian [40]	
Instrument	Laser scanner		Caliper		Caliper		Caliper		Caliper		Caliper		Caliper		Contact digitizer	
Age (years)	18–30		18–25		18–30		18–30		18–30		18–30		19–25		18–30	
N	75		80		30		30		30		36		109		66	
mm	Mean	SD	Mean	SD	Mean	SD	Mean	SD	Mean	SD	Mean	SD	Mean	SD	Mean	SD
ch-ch	55.9	4.9	54.6	4.1	54.4	3.0	48.3	3.7	56.2	5.3	55.9	3.3	54.5	3.0	51.9	3.8
cph-cph	13.9	3.4	13.0	2.1									10.4	1.4	12.4	2.1
ls-li	20.7	3.3	27.4 ^§^	2.1									17.3 ^§^	1.4	15.2	3.4
ls-sto	10.9	2.3	13.6	2.1									8.0	1.4	6.7	2.3
sto-li	10.8	2.3	13.8	2.1									9.3	1.6	8.8	3.0
sn-sl	42.9	4.5	46.2 ^#^	2.5							48.0 ^#^	3.1	42.0 ^#^	4.6	42.8	3.8

AA: African American; NAW: North American White; SD: standard deviation; ^§^: sum of ls-sto and sto-li; ^#^: sum of sn-sto and sto-sl.

## Data Availability

The data that support the findings of this study are available on request from the corresponding author. The data are not publicly available due to privacy and ethical restrictions.
